# Nocardia farcinica Brain Abscess Mimicking Acute Ischemic Stroke in an Immunocompetent Elderly Patient: A Case Report

**DOI:** 10.7759/cureus.101988

**Published:** 2026-01-21

**Authors:** Inês Fiúza M. Rua, Sérgio Cabaço, Diogo D Ramos, Lilian C Farias, Ana Margarida Serrano

**Affiliations:** 1 Internal Medicine, Unidade Local de Saúde São José, Lisbon, PRT; 2 Infectious Diseases, Unidade Local de Saúde São José, Lisbon, PRT

**Keywords:** brain abscess, central nervous system, central nervous system infection, cns nocardia, immuno-competent, infection, nocardia, nocardia farcinica, nocardiosis

## Abstract

Central nervous system (CNS) infections caused by *Nocardia* species are rare but potentially life-threatening. Even more unusual is *Nocardia* infection in immunocompetent patients. Most patients initially present with a primary respiratory or cutaneous infection that may progress to disseminated disease involving the CNS; however, primary CNS infection is rare. *Nocardia farcinica* is frequently associated with brain abscesses. We report the case of an 81-year-old immunocompetent man who presented with acute focal neurological deficits and seizures, initially managed as an ischemic stroke. Subsequent clinical deterioration and radiological progression led to suspicion of an alternative diagnosis, prompting initiation of empirical broad-spectrum antibiotic therapy for a presumed CNS infection. Definitive identification of *N. farcinica* was achieved only after neurosurgical biopsy, allowing targeted antimicrobial therapy with subsequent clinical and radiological improvement. This case highlights the diagnostic challenges posed by nocardial brain abscesses, their ability to mimic acute cerebrovascular events, and the importance of early surgical diagnosis and prolonged targeted antimicrobial therapy.

## Introduction

*Nocardia* is an opportunistic pathogen that typically causes disease in immunocompromised individuals; although commonly found in the environment, infection in immunocompetent hosts is uncommon, with a reported incidence of approximately 0.4 cases per 100,000 individuals [1-5].

The respiratory tract is the most frequently affected site, accounting for approximately 70% of cases, with infection attributed to inhalation of contaminated particles [6]. Primary cutaneous infection, representing about 25% of cases, is associated with injection drug use and traumatic skin exposure [7]. Most patients initially present with respiratory or cutaneous infection. In cases of systemic infection, hematogenous dissemination to multiple organs may occur, most notably to the central nervous system (CNS), which is involved in approximately 40% of cases, making it the most common site of secondary infection [3,6]. However, CNS involvement in patients without prior respiratory or cutaneous infection is rare [1,2,5,8-10].

The main risk factors for CNS infection caused by *Nocardia* include conditions associated with impaired cell-mediated immunity, particularly prolonged or high-dose corticosteroid therapy, autoimmune diseases, solid organ transplantation, malignancy, HIV infection, diabetes mellitus, chronic kidney disease, and underlying chronic lung disease [1,2,10,11]. Lymphopenia (<1.0 × 10⁹/L) has also been identified as a laboratory marker associated with an increased risk of disseminated disease, including CNS involvement [12].

*Nocardia farcinica* is one of the species most frequently associated with brain abscesses and is characterized by an aggressive pathogenic profile, a high propensity for hematogenous dissemination, and significant antimicrobial resistance [1,2,8-10].

The clinical presentation of CNS nocardiosis is typically insidious and may include focal neurological deficits, seizures, behavioral changes, or signs of intracranial hypertension. The diagnostic process is often challenging, as the absence of specific clinical findings frequently leads to an initial misdiagnosis and subsequent diagnostic delay [2,11]. Furthermore, neuroimaging findings such as multiple abscesses or space-occupying lesions may mimic neoplasms or other infectious processes [1,8,10,11,13,14].

We report a case of *N. farcinica* brain abscess in an immunocompetent elderly patient, without associated risk factors or prior respiratory or cutaneous infection, illustrating the diagnostic and therapeutic challenges of this rare entity.

## Case presentation

An 81-year-old man, previously independent, with a medical history of essential hypertension, permanent atrial fibrillation, and dyslipidemia, presented to the emergency department. His regular medication included amlodipine, losartan/hydrochlorothiazide, and apixaban. He had no known drug allergies and no relevant epidemiological history.

He was admitted due to the sudden onset of confusion, disorganized speech, dysarthria, and decreased strength in the left upper limb. Upon admission, he developed a generalized tonic-clonic seizure, which was controlled with intravenous diazepam and levetiracetam, followed by gradual recovery of consciousness.

Neurological examination revealed an alert patient with left central facial paresis, moderate dysarthria consistent with a motor speech disturbance (National Institutes of Health Stroke Scale (NIHSS) item 10 score: 1), left hemiparesis (Medical Research Council grade 3 in the left upper limb and grade 4 in the left lower limb), anosognosia, asomatognosia, left hemisensory loss to pain, and an indifferent plantar reflex on the left side (NIHSS score: 12; Glasgow Coma Scale: 15). The remainder of the physical examination was unremarkable; the patient was afebrile and hemodynamically stable.

Given the clinical presentation, the acute stroke protocol was activated. Initial non-contrast cranioencephalic computed tomography (CT) revealed a right frontoparietal and insular hypodense parenchymal area, predominantly subcortical with partial cortical involvement. Associated findings included mass effect, sulcal effacement, and mild compression of the right lateral ventricle. CT angiography demonstrated patency of the supra-aortic and intracranial arterial vessels. Laboratory studies, including complete blood count, renal and liver function tests, inflammatory markers, and electrolytes, were within normal limits.

Thrombolytic therapy was contraindicated due to ongoing anticoagulation, and there was no indication for mechanical thrombectomy. Secondary stroke prevention with acetylsalicylic acid and a high-intensity statin was initiated.

**Figure 1:**
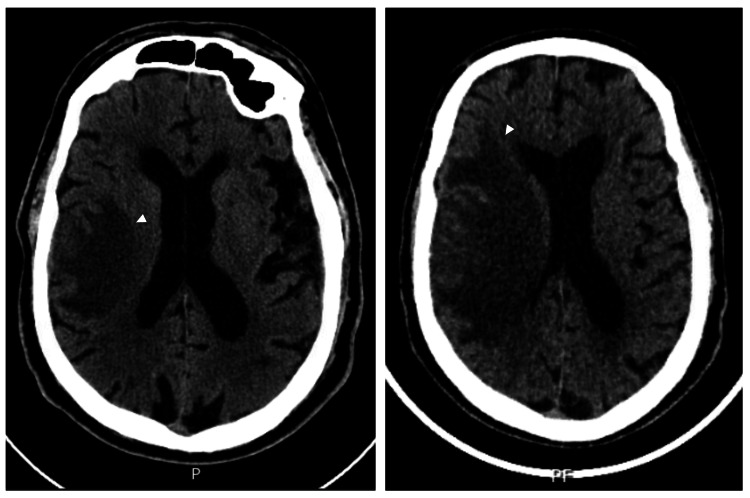
Cranioencephalic computed tomography (CT) findings during initial evaluation. Left: Non-contrast cranioencephalic CT at admission demonstrating a right frontoparietal hypodense lesion (white arrowhead) with associated mass effect, initially interpreted as acute ischemic stroke. Right: Repeat non-contrast cranioencephalic CT performed due to neurological deterioration, showing lesion progression (white arrowhead) with increased mass effect and surrounding vasogenic edema, raising suspicion of a space-occupying lesion.

A follow-up CT scan at 24 hours showed slight progression of hypodensity without hemorrhagic transformation, maintaining the working diagnosis of ischemic stroke, although alternative etiologies could not be excluded (Figure 1). The patient was admitted for monitoring and etiological investigation of a presumed right middle cerebral artery ischemic stroke.

On the fourth day of hospitalization, neurological deterioration prompted repeat CT imaging, raising suspicion of a space-occupying lesion with associated vasogenic edema (Figure 1).

**Figure 2:**
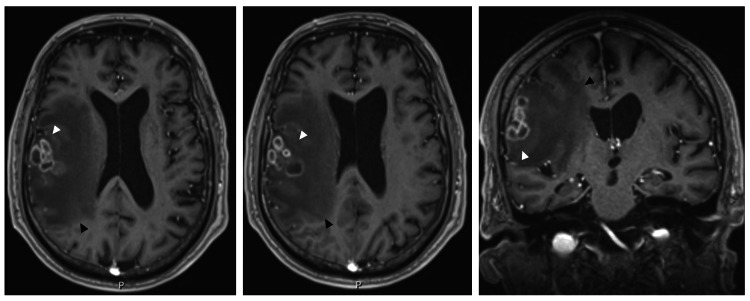
Cranioencephalic MRI. Left and center: Axial contrast-enhanced MRI images demonstrating a right frontal intra-axial cortico-subcortical lesion composed of a confluent conglomerate of rounded lesions measuring approximately 35 mm (white arrowheads), associated with extensive surrounding vasogenic edema (black arrowheads) and mass effect. Right: Coronal contrast-enhanced MRI image further illustrating the multiloculated appearance and intra-axial distribution of the lesion. Overall imaging features were suggestive of a central nervous system abscess. MRI, magnetic resonance imaging.

Brain magnetic resonance imaging (MRI) was performed, revealing a right frontal intra-axial cortico-subcortical expansile lesion composed of a confluent conglomerate of rounded lesions measuring approximately 35 mm, associated with extensive vasogenic edema. CNS abscesses were considered the most likely diagnosis, while a neoplastic etiology was deemed unlikely (Figure 2).

Empirical antibiotic therapy with meningeal doses of ceftriaxone, metronidazole, and ampicillin, along with dexamethasone, was initiated, resulting in significant initial clinical improvement. Cerebrospinal fluid (CSF) analysis was unremarkable, with negative microbiological, mycological, and mycobacteriological studies (Table 1).

**Table 1:** Cerebrospinal fluid analysis.

Laboratory Parameter	Results	Reference Range Values
Macroscopic examination	Clear and colorless	-
Leukocytes (/µL)	10	0-5
Mononuclear cells (/µL)	9	
Polymorphonuclear cells (/µL)	1	
Erythrocytes (x10^3^/µL)	Rare	-
Glucose (mg/dL)	102.7	40-70
Proteins (mg/dL)	58	15-45
Lactate dehydrogenase (U/L)	26	<40
Adenosine deaminase (ADA) (U/L)	0.5	<9
Anti-Borrelia antibodies (IgG+IgM)	Negative	-
*Toxoplasma gondii* (PCR)	Not detected	-
*Cryptococcus neoformans* antigen	Negative	-
Microbiological culture	Sterile	-
Mycological examination	Negative	-
Nucleic acid amplification test (NAAT) for *Mycobacterium tuberculosis*	Negative	-
Mycobacterial culture (Lowenstein-Jensen and Mycobacteria growth indicator tube)	Negative	-

During hospitalization, an extensive infectious workup was performed, including serological testing for HIV, syphilis, *Coxiella burnetii*, and *Borrelia burgdorferi*, all of which were negative. Serial blood cultures, including prolonged incubation for fastidious organisms and mycobacteria, showed no microbial growth (Table 2).

**Table 2:** Investigation for infectious agents.

Laboratory Parameter	Results
Anti-HIV 1+2 antibodies	Negative
Syphilis (Venereal Disease Research Laboratory - VDRL)	Negative
Anti-*Coxiella burnetii* (IgG + IgM – Phase I and Phase II)	Negative
Anti-*Borrelia burgdorferi* (IgG + IgM)	Negative
Blood culture (5-day incubation) – Day 12	Sterile
Blood cultures – mycobacteriological examination (42-day incubation) – Day 12	Sterile
Blood culture (5-day incubation) – Day 13	Sterile
Blood culture (21-day incubation) – Day 18	Sterile
Blood cultures (21-day incubation) – Day 22	Sterile

Transesophageal echocardiography was performed to exclude an embolic infectious source and revealed a small filamentous structure adjacent to the anterior mitral valve leaflet, considered a doubtful vegetation. Further evaluation with a positron emission tomography (PET) scan was recommended if clinical suspicion persisted. Thoraco-abdominopelvic CT and thyroid ultrasound were also performed to exclude a primary malignancy with CNS metastasis, both without abnormal findings.

MRI performed on day 14 of antibiotic therapy demonstrated an increase in lesion size, with formation of a conglomerate measuring approximately 50 mm, with partial improvement of vasogenic edema and mass effect. In the setting of radiological progression and absence of pathogen isolation, vancomycin was added to the antimicrobial regimen, and a PET scan was performed, which did not reveal any active extracranial infectious focus.

**Figure 3:**
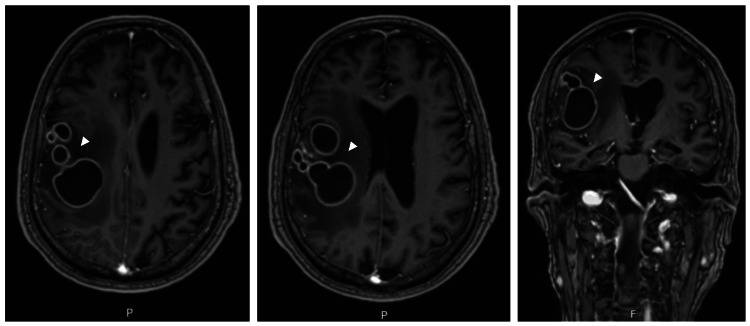
Follow-up cranioencephalic MRI performed after antibiotic therapy. Left and center: Axial contrast-enhanced MRI images demonstrating progression in the size of multiple contiguous right frontal intra-axial lesions (white arrowheads), despite partial improvement of surrounding vasogenic edema. Right: Coronal contrast-enhanced MRI image further illustrating lesion enlargement and persistent mass effect. These findings indicated failure of conservative medical management and prompted neurosurgical intervention. MRI, magnetic resonance imaging.

A subsequent MRI on day 27 showed continued progression, prompting neurosurgical intervention due to failure of conservative medical management (Figure 3).

A right frontoparietal craniotomy with aspirative biopsy was performed due to lesion progression, multiloculated morphology, and significant mass effect, in order to achieve complete source control and effective decompression, and microbiological culture of the surgical specimen confirmed *N. farcinica* as the causative pathogen. Following multidisciplinary discussion, targeted intravenous therapy with trimethoprim-sulfamethoxazole (15 mg/kg/day of the trimethoprim component, divided into three doses) and imipenem (500 mg every 6 hours) was initiated.

**Figure 4:**
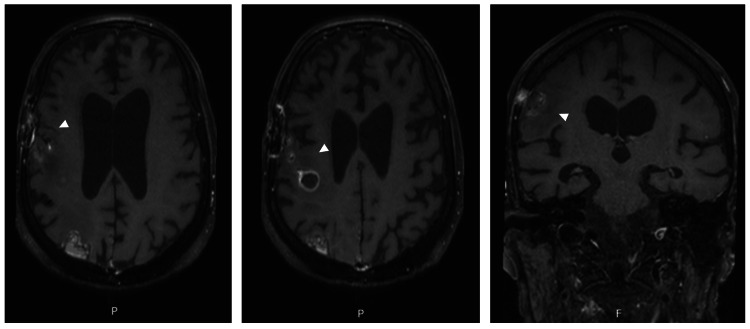
Postoperative follow-up cranioencephalic MRI. Left and center: Axial contrast-enhanced MRI images obtained three weeks after surgery and initiation of targeted anti-nocardial therapy, demonstrating progressive radiological improvement, with reduction in the size of the right frontal intra-axial lesions and surrounding vasogenic edema (white arrowheads), following neurosurgical intervention and initiation of targeted antimicrobial therapy. Right: Coronal contrast-enhanced MRI image further illustrating lesion regression and decreased mass effect. MRI, magnetic resonance imaging.

Serial postoperative imaging demonstrated progressive radiological improvement, with a reduction in lesion size and surrounding edema (Figure 4).

After three weeks of intravenous therapy, given sustained clinical and radiological improvement, treatment was transitioned to oral trimethoprim-sulfamethoxazole (800/160 mg every 8 hours) and amoxicillin/clavulanic acid (875/125 mg every 12 hours), dexamethasone was gradually tapered, and the patient was discharged to a rehabilitation unit.

Despite a favorable neurological recovery, two months after admission to the rehabilitation unit, the patient developed a healthcare-associated respiratory infection, progressing to septic shock requiring intensive care admission, and ultimately resulting in death during hospitalization.

## Discussion

Brain abscesses caused by *N. farcinica *in immunocompetent individuals are rare but associated with high morbidity and mortality [1,2,9,10]. This case illustrates several classical diagnostic challenges of CNS nocardiosis [14]. Initial misdiagnosis as ischemic stroke, as observed in this patient, is a common pitfall due to the nonspecific and often progressive neurological presentation, which may significantly delay appropriate therapy [15]. Additionally, neuroimaging findings of ring-enhancing lesions, although suggestive of abscess, are not pathognomonic. These findings may overlap with high-grade neoplasms and other infectious processes, complicating definitive radiological diagnosis [16,17].

As previously described, most cases present initially with respiratory or cutaneous infection followed by hematogenous dissemination to the CNS [6,7]. In this case, the patient was otherwise healthy, without clinical evidence of an extracranial infectious focus, representing a rare presentation of primary CNS nocardiosis, which occurs in fewer than 10% of cases [14].

Definitive diagnosis was established only after surgical excision, which confirmed a pyogenic abscess and enabled isolation of *N. farcinica*. This case underscores the limited diagnostic value of CSF analysis in brain abscesses, as negative CSF cultures are common in the absence of meningitis [14].

Management consists of first-line antimicrobial therapy combined with aspirative biopsy or decompressive craniotomy, both to establish diagnosis and to achieve source control and reduction of mass effect [1,8,9,13,14]. Trimethoprim-sulfamethoxazole remains the cornerstone of therapy, with susceptibility rates exceeding 97% for *N. farcinica* [2,11,13]. In severe, disseminated, or multifocal disease, combination therapy with imipenem or amikacin is recommended [1,2,8-11,13].

Treatment duration should be prolonged, typically ranging from six to 12 months, particularly in *N. farcinica* brain abscesses, due to the organism’s aggressive pathogenic profile and high relapse risk [1,7,10,18]. Transition to oral therapy should be carefully considered, based on clinical stability, radiological improvement, and absence of complications [9,19,20]. When susceptibility testing is unavailable, preferred oral agents include trimethoprim-sulfamethoxazole, linezolid, minocycline, moxifloxacin, and amoxicillin [19,20].

Despite appropriate management, mortality remains substantial, particularly in *N. farcinica* infections, even among immunocompetent patients. Reported treatment success rates range from 70% to 90%, with mortality between 10% and 30% and relapse rates below 5% when therapy is adequate and prolonged [1,8,10]. A multidisciplinary approach, early diagnosis, prompt initiation of targeted antimicrobial therapy, careful transition to oral agents, and close clinical and radiological monitoring are essential to optimize outcomes [1-4,8-11,13-20].

## Conclusions

Brain abscesses caused by *N. farcinica* should be considered in the differential diagnosis of intracranial space-occupying lesions, even in immunocompetent patients. This case highlights the diagnostic challenges posed by nonspecific clinical and radiological presentations that may initially mimic acute cerebrovascular events and delay appropriate treatment.

Optimal management requires an aggressive, multidisciplinary approach coordinated by internal medicine, integrating timely neurosurgical intervention for effective source control and definitive microbiological diagnosis, along with close collaboration with infectious disease specialists, radiologists, and microbiologists to guide targeted antimicrobial therapy and monitor response. In *N. farcinica* brain abscesses, antimicrobial treatment typically involves an initial intravenous phase followed by prolonged oral therapy extending over several months to minimize the risk of relapse.

Prognosis largely depends on early diagnosis, adequate source control, and adherence to prolonged targeted antimicrobial therapy; however, despite appropriate management, morbidity and mortality remain substantial, particularly in elderly patients.
